# Serum KL-6 and the mortality of patients with connective tissue disease-associated interstitial lung disease: A meta-analysis

**DOI:** 10.17305/bb.2024.10368

**Published:** 2024-10-01

**Authors:** Mei Hong, Xue Yin, Wenmei Yan, Wei Guo, Hongmei Liu, Haisheng Yang

**Affiliations:** 1 Department of Dermatology, The People’s Hospital of Wuhai, Wuhai, China; 2 Department of Pulmonary and Critical Care Medicine, The People’s Hospital of Wuhai, Wuhai, China

**Keywords:** Interstitial lung disease (ILD), connective tissue disease (CTD), Krebs von den Lungen-6 (KL-6), mortality, meta-analysis

## Abstract

Connective tissue disease-associated interstitial lung disease (CTD-ILD) is an important underlying cause of morbidity and mortality in patients with connective tissue disease (CTD). Serum Krebs von den Lungen-6 (KL-6) is an immune factor that has been related to the severity of interstitial lung disease (ILD). This systematic review and meta-analysis aimed to evaluate the association between serum KL-6 and mortality of patients with CTD-ILD. Longitudinal studies relevant to the aim of the meta-analysis were retrieved by search of electronic databases including PubMed, Web of Science, and Embase. A random-effects model was used to combine the data by incorporating the influence of between-study heterogeneity. Fifteen cohorts involving 1737 patients with CTD-ILD were included. During a mean follow-up of 35.3 months, 430 (24.8%) patients died. Compared to those with a lower KL-6 at admission, patients with a higher KL-6 were associated with a higher mortality risk during follow-up (risk ratio: 2.18, 95% confidence interval: 1.66–2.87, *P* < 0.001; *I*^2^ ═ 20%). Subgroup analysis showed a significant association in studies from Asian countries, but not in those from non-Asian countries; in studies with a cutoff of KL-6 derived in receiver operating characteristic (ROC) curve analysis, but not in those derived from other methods; in studies with multivariate analysis, but not in those with univariate analysis (*P* for subgroup difference all < 0.05). The association was not significantly affected by different CTDs or methods for measuring serum KL-6. In conclusion, a high serum KL-6 may be a risk factor for increased mortality in patients with CTD-ILD.

## Introduction

Connective tissue diseases (CTDs) encompass a diverse group of conditions sharing common pathophysiological mechanisms involving autoimmunity and immune-mediated organ dysfunction [1]. Interstitial lung disease (ILD) is a prevalent pulmonary manifestation observed in various CTDs, including polymyositis/dermatomyositis (PM/DM), rheumatoid arthritis (RA), systemic lupus erythematosus (SLE), systemic sclerosis (SSc), Sjogren’s syndrome (SS), and undifferentiated CTDs [2, 3]. Pathologically, ILD adversely affects the interstitium, the tissue layer surrounding the alveoli, potentially leading to alterations in alveolar and airway architecture [4, 5]. In patients with CTDs, ILD may be diagnosed concurrently with or subsequent to the CTD diagnosis, known as CTD-associated ILD (CTD-ILD) [6]. Despite the variability in disease progression, CTD-ILD is recognized as a significant contributor to morbidity and mortality in individuals with CTD [7]. However, there is currently no evidence-based gold standard for managing CTD patients [8, 9], highlighting the importance of the research for discovering potential mechanisms underlying the pathogenesis of CTD-ILD.

Krebs von den Lungen-6 (KL-6) is a high-molecular-weight glycoprotein encoded by the MUC1 gene, primarily located on the cell surface of type II alveolar epithelial cells and bronchial epithelial cells [10]. Elevated serum levels of KL-6 have been identified as a biomarker for alveolar epithelial proliferation and injury in various respiratory conditions [10], including acute respiratory distress syndrome [11], hypersensitivity pneumonia [12], idiopathic interstitial pneumonia [13], pulmonary sarcoidosis [14], coronavirus disease 2019 (COVID-19) [15], and CTD-ILD [16]. Furthermore, several studies have indicated the potential predictive value of serum KL-6 in determining the prognosis of individuals with severe lung conditions, including idiopathic pulmonary fibrosis [17], and COVID-19 [18]. Nevertheless, research examining the correlation between serum KL-6 levels and mortality risk in patients with CTD-ILD has yielded conflicting results [19]. While some studies have suggested that elevated baseline levels of serum KL-6 may be indicative of an increased mortality risk in individuals with CTD-ILD [20–26], others have not found a significant association [27–30]. Therefore, in this study, we performed a meta-analysis to systematically evaluate the association between serum KL-6 and mortality of patients with CTD-ILD.

## Materials and methods

The Preferred Reporting Items for Systematic Reviews and Meta-Analyses (PRISMA) statement (2020) [31, 32] was followed in this study. The Cochrane Handbook [33] for systematic review and meta-analysis was referenced throughout the study.

### Inclusion and exclusion criteria

Inclusion criteria were:

(1) Studies with longitudinal follow-up, such as prospective/retrospective cohort studies, nested case-control studies, and post-hoc analysis of clinical studies, presented in full-length articles.

(2) Studies that included adult patients with a confirmed diagnosis of CTD-ILD without limitations of the associated CTDs.

(3) Serum KL-6 was measured at admission or enrollment, and patients with a higher serum level of KL-6 were considered as exposure. The cutoff for defining a higher serum level of KL-6 was consistent with the cutoff used in the original studies.

(4) Patients with a lower serum level of KL-6 at baseline were considered as controls.

(5) Reported the outcome of all-cause mortality during follow-up, compared between CTD-ILD patients with higher vs lower serum levels of KL-6 at baseline.

Reviews, meta-analyses, cross-sectional studies, studies with serum KL-6 analyzed as continuous variables, or studies without the outcome of interest were excluded. For studies with potentially overlapped patient populations, the one with the largest sample size was included in the meta-analysis.

### Literature analysis

Three major electronic databases, including PubMed, Web of Science, and Embase, were used for the literature search with a predefined combined search term including (“interstitial lung disease” OR “ILD” OR “interstitial pneumonia”) AND (“KL-6” OR “Krebs von den Lungen-6” OR “KL 6”) and (“mortality” OR “death” OR “survival” OR “prognosis”). Only studies with human subjects and published in peer-reviewed journals in English were included. A second-round check-up for the references of the relevant articles was also conducted. The final database search was achieved on December 11, 2023.

### Data collection and quality assessment

Two independent authors conducted a literature search and analysis, data collection, and study quality assessment separately. If discrepancies were encountered, the corresponding author joined the discussion for final judgment. Data on study information, study design, diagnosis, demographic factors of the patients, timing and methods for measuring serum KL-6, methods for developing the cutoff of KL-6, follow-up duration, number of patients who died during follow-up, and variables adjusted in the regression model for the association between KL-6 and mortality were collected. Study quality assessment was achieved via the Newcastle–Ottawa Scale [34] with scoring regarding the criteria for participant selection, comparability of the groups, and the validity of the outcomes. The scale ranged between 1 and 9 stars, with a larger number of stars presenting higher study quality.

### Ethical statement

Ethical approval was not required for this study in accordance with local/national guidelines. Written informed consent to participate in the study was also not required in accordance with local/national guidelines.

### Statistical analysis

The relative risk of all-cause mortality, compared between patients with CTD-ILD with a higher vs a lower serum KL-6 at baseline, was presented as risk ratios (RRs) as well as their confidence intervals (CIs). Data for outcomes adjusted for the highest number of variables were used for the meta-analysis. Where the odds ratios (ORs) were presented, data were converted to RRs for the meta-analysis (RR=OR/([1-pRef]+[pRef×OR]), where pRef is the prevalence of the outcome in the reference group (lower KL-6 group) [35]. Using the 95% CIs or *P* values, data of RRs and the standard errors (SEs) could be calculated, and a subsequent logarithmical transformation was conducted to keep stabilized variance and normalized distribution [33]. Between study heterogeneity was estimated with the Cochrane *Q* test and the *I*^2^ statistic [36, 37], with *I*^2^ > 50% reflecting the significant heterogeneity. A random-effect model was applied to combine the results by incorporating the influence of heterogeneity [33]. Sensitivity analysis by excluding one study at a time was used to evaluate the robustness of the finding [33]. Predefined subgroup analyses were performed to evaluate the potential influences of study characteristics on the association between serum KL-6 and mortality, including study country, different CTD diseases, methods for measuring serum KL-6, methods for deriving the cutoffs of KL-6, analytic models for the association between serum KL-6 and mortality (univariate or multivariate regression model), and mean follow-up durations. By construction of the funnel plots, the publication bias was estimated based on the visual judgment of the symmetry of the plots, supplemented with Egger’s regression asymmetry test [38]. A *P* < 0.05 reflects statistical significance. The RevMan (version 5.1; Cochrane Collaboration, Oxford, UK) and Stata (version 12.0; Stata Corporation, College Station, TX, USA) software packages were applied for these analyses.

## Results

### Study inclusion

**Figure 1.**
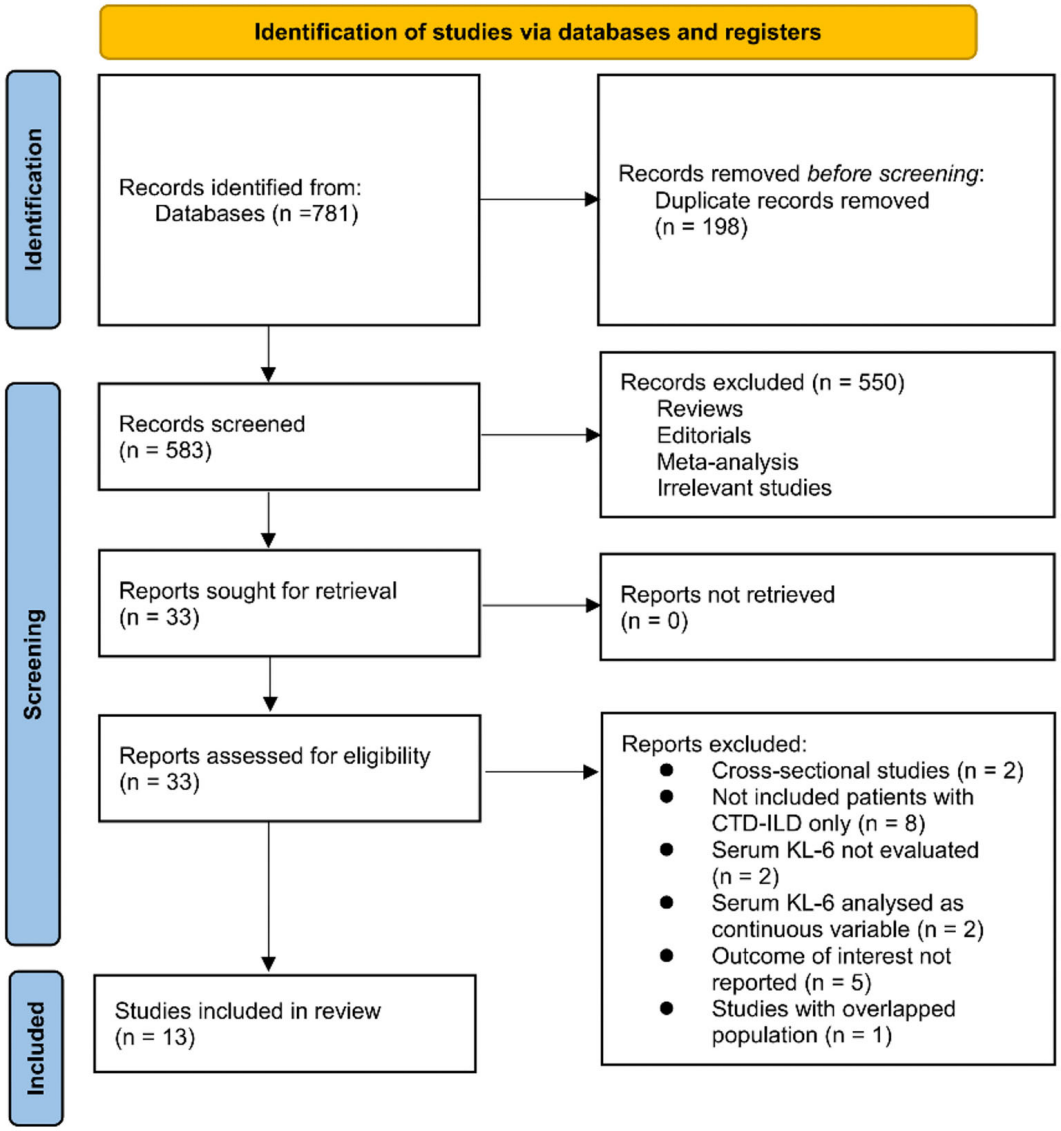
**Process of literature search and study identification.** CTD-ILD: Connective tissue disease-associated interstitial lung disease; KL-6: Krebs von den Lungen-6.

The process for identifying relevant studies for inclusion in the meta-analysis is presented in Figure 1. In brief, 781 potentially relevant records were obtained after comprehensive searches of the three databases, and 198 of them were excluded due to duplication. Subsequently, a screening via considering the titles and abstracts of the remaining records further led to the exclusion of 550 more studies, mostly because they were not related to the aim of the meta-analysis. Accordingly, the full texts of the 33 remaining records were read by two independent authors, and 20 of them were further removed for various reasons, as listed in Figure 1. Finally, 13 records remained and were considered to be suitable for the subsequent quantitative analyses [20–30, 39, 40].

### Overview of the studies’ characteristics

Table 1 presents the summarized characteristics of the included studies. Because one study reported outcomes according to the different treatments of the patients [28], and another study included two cohorts with different study designs (retrospective and prospective) [40], these cohorts were included independently, making 15 cohorts available for meta-analysis. These studies were published between 2013 and 2023, and performed in Japan, the United States, China, Korea, and Italy. Patients with various CTDs were included, such as PM/DM, RA, SLE, SSc, SS, and undifferentiated CTD, all with CTD-ILD. Overall, 15 cohorts involving 1737 patients with CTD-ILD were included [20–30, 39, 40]. Serum KL-6 was measured at admission/enrollment with the enzyme immunoassay (EIA), the enzyme-linked immunosorbent assay (ELISA), and the immunoturbidimetric assay (ITA). The cutoff of KL-6 was derived via receiver operating characteristic (ROC) curve analysis in 12 cohorts [20–26, 29, 30, 39, 40]; whereas in the other three cohorts, the cutoff of KL-6 was defined via the upper limit of the normal value [27] and the medians [28]. The follow-up durations were 4–101 months. During a mean follow-up of 35.3 months, 430 (24.8%) patients died. The univariate regression analysis was performed in four cohorts [21, 27, 28] in estimating the association between KL-6 and mortality, while the multivariate regression analysis was performed in the other 11 cohorts [20, 22–26, 29, 30, 39, 40] with adjustment at least for age and sex of the patients. The NOS of the included studies were six to eight stars, suggesting overall moderate to good study quality (Table 2).

**Table 1:** Study characteristics

**Study**	**Country**	**Design**	**Diagnosis**	**No. of patients included**	**Mean age (years)**	**Female (%)**	**Timing of KL-6 assessment**	**Methods for measuring KL-6**	**Serum KL-6 cutoff methods**	**Serum KL-6 cutoff value (U/mL)**	**Follow-up duration (months)**	**No. of patients died**	**Variables adjusted**
Arai, 2013	Japan	RC	PM/DM+ILD	50	54.4	74	At admission	EIA	Normal value upper limits	500	12	7	None
Volkmann, 2019 CYC	USA	Post-hoc	SSc+ILD	71	52.3	77.5	At enrollment	ELISA	Median	330.7	48	16	None
Volkmann, 2019 MMF	USA	Post-hoc	SSc+ILD	62	52.9	71	At enrollment	ELISA	Median	330.7	48	14	None
Ye, 2019	China	RC	Anti-MDA5+ DM and ILD	128	50.9	94	At diagnosis	EIA	ROC curve analysis	792	12	18	None
Fujisawa, 2019	Japan	RC	Anti-MDA5+ DM and ILD	30	54	77	At admission	ELISA	ROC curve analysis	720	4	10	Age, sex, ferritin, and chitotriosidase
Kamiya, 2019	Japan	RC	SS+ILD	99	68	72.7	At admission	ELISA	ROC curve analysis	800	60	21	Age and sex
Kim, 2020	Korea	RC	RA+ILD	84	61.4	54.8	At admission	ITA	ROC curve analysis	685	61	33	Age, sex, lower FVC, and UIP pattern
Gono, 2021	Japan	RC	PM/DM+ILD	497	57	66	At admission	ELISA	ROC curve analysis	1000	20	93	Age, sex, CRP, and treatment
Yang, 2021	China	RC	Anti-MDA5+ DM/PM and ILD	90	51.9	63.3	At admission	ELISA	ROC curve analysis	1600	6	22	Age, sex, ferritin, HRCT score, and treatment
Stock, 2021 RC	Italy	RC	SSc+ILD	189	49.1	77.3	At admission	ITA	ROC curve analysis	1472	101	92	Age, sex, ethnicity, CPI, and smoking history
Stock, 2021 PC	Italy	PC	SSc+ILD	118	56.4	76.3	At admission	ITA	ROC curve analysis	NR	33	17	Age, sex, ethnicity, CPI, and smoking history
Zhu, 2022	China	RC	Anti-MDA5+DM and ILD	21	55.3	61.9	At enrollment	ITA	ROC curve analysis	NR	12	8	Age, sex, ferritin, and ESR
Meng, 2022	China	RC	CTD (RA/SS/UCTD)+ILD	55	67	74.5	At enrollment	EIA	ROC curve analysis	964.5	23	29	Age, sex, smoking, FVC, and GAP index
Kim, 2022	Korea	RC	SS+ILD	46	59.4	82.6	At admission	EIA	ROC curve analysis	NR	69	12	Age, sex, smoking, FVC, and UIP pattern
Lee, 2023	Korea	RC	CTD (RA/DM/PM/SS/SSc/SLE/UCTD)+ILD	197	64	60.1	At admission	ELISA	ROC curve analysis	1000	17.4	38	Age, sex, albumin, pulmonary function, and UIP pattern

KL-6: Krebs von den Lungen-6; ILD: Interstitial lung disease; PM/DM: Polymyositis/dermatomyositis; EIA: Enzyme immunoassay; SSc: Systemic sclerosis; anti-MDA5+ DM: Ant-MDA5 antibody-positive dermatomyositis; ELISA: Enzyme-linked immunosorbent assay; HRCT: High-resolution computed tomography; RA: Rheumatoid arthritis; ITA: Immunoturbidimetric assay; FVC: Lower forced vital capacity; UIP: Usual interstitial pneumonia; CPI: Composite physiological index; CTD: Connective tissue disease; UCTD: Undifferentiated connective tissue disease; GAP: Gender-Age-Physiology; SS: Sjogren’s syndrome; SLE: Systemic lupus erythematosus; ROC: Receiver operating characteristic; ESR: Erythrocyte sedimentation rate; MMF: Mycophenolate mofetil; CYC: Cyclophosphamide; RC: Randomized controlled; PC: Prospective cohort.

**Table 2:** Study quality assessment

**Study**	**Representativeness of the exposed cohort**	**Selection of the non-exposed cohort**	**Ascertainment of exposure**	**Outcome not present at baseline**	**Control for age and sex**	**Control for other confounding factors**	**Assessment of outcome**	**Long enough follow-up duration**	**Adequacy of follow-up of cohorts**	**Total points**
**Arai, 2013**	1	1	1	1	0	0	1	1	1	7
**Volkmann, 2019 CYC**	0	1	1	1	0	0	1	1	1	6
**Volkmann, 2019 MMF**	0	1	1	1	0	0	1	1	1	6
**Ye, 2019**	0	1	1	1	0	0	1	1	1	6
**Fujisawa, 2019**	0	1	1	1	1	1	1	0	1	7
**Kamiya, 2019**	1	1	1	1	1	0	1	1	1	8
**Kim, 2020**	0	1	1	1	1	1	1	1	1	8
**Gono, 2021**	0	1	1	1	1	1	1	1	1	8
**Yang, 2021**	0	1	1	1	1	1	1	1	1	8
**Stock, 2021 RC**	0	1	1	1	1	1	1	1	1	8
**Stock, 2021 PC**	1	1	0	1	1	1	1	1	1	8
**Zhu, 2022**	0	1	1	1	1	1	1	1	1	8
**Meng, 2022**	0	1	1	1	1	1	1	1	1	8
**Kim, 2022**	0	1	1	1	1	1	1	1	1	8
**Lee, 2023**	0	1	1	1	1	1	1	1	1	8

MMF: Mycophenolate mofetil; CYC: Cyclophosphamide; RC: Randomized controlled; PC: Prospective cohort.

**Figure 2.**
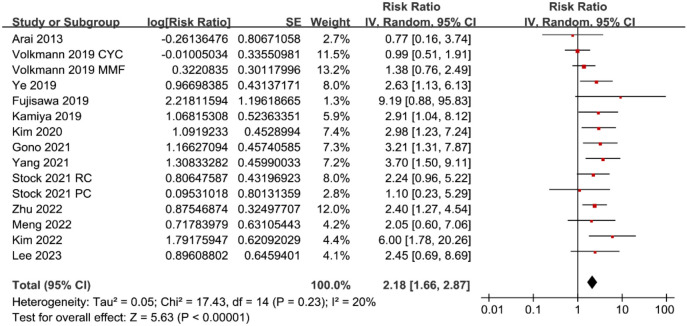
**Forest plots for the meta-analysis of the association between serum KL-6 and mortality risk in patients with CTD-ILD.** CTD-ILD: Connective tissue disease-associated interstitial lung disease; KL-6: Krebs von den Lungen-6; CI: Confidence interval.

**Figure 3.**
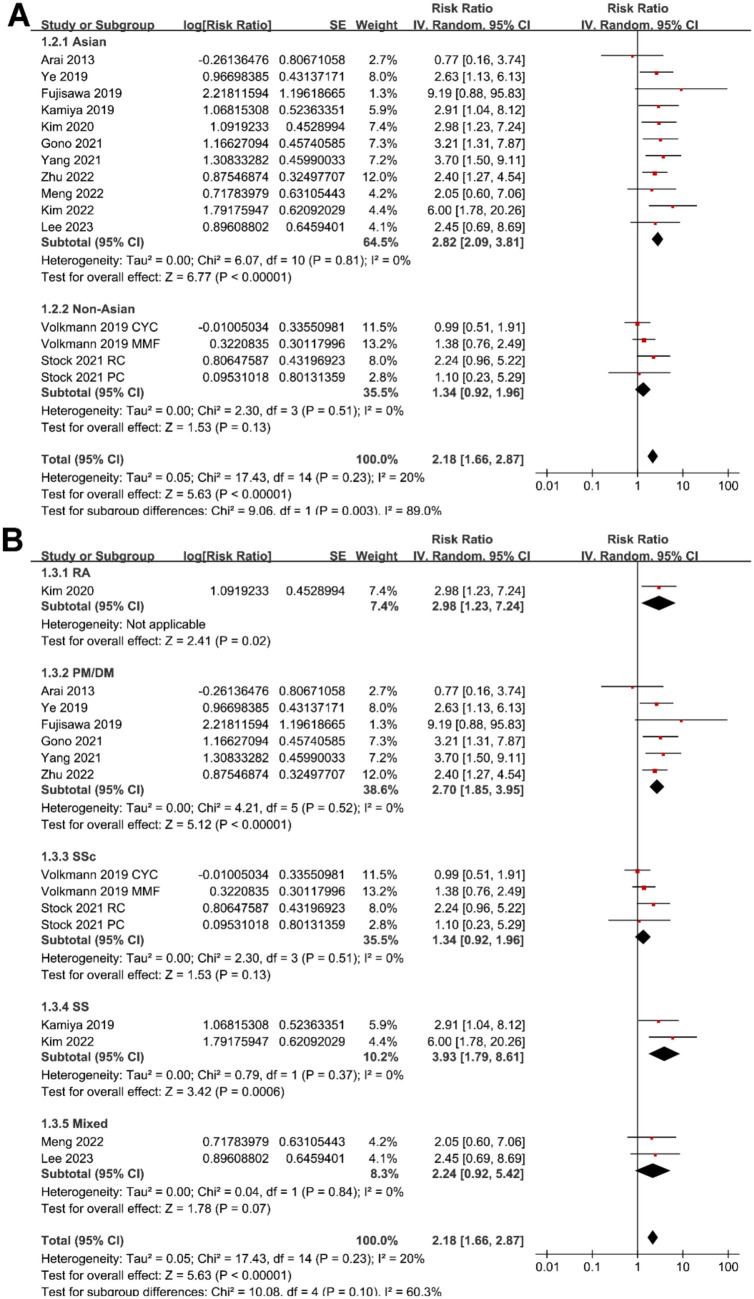
**Forest plots for the subgroup analyses of the association between serum KL-6 and mortality risk in patients with CTD-ILD.** (A) Subgroup analysis according to study country and (B) different CTDs. CTD-ILD: Connective tissue disease-associated interstitial lung disease; KL-6: Krebs von den Lungen-6; CI: Confidence interval; SE: Standard error.

**Figure 4.**
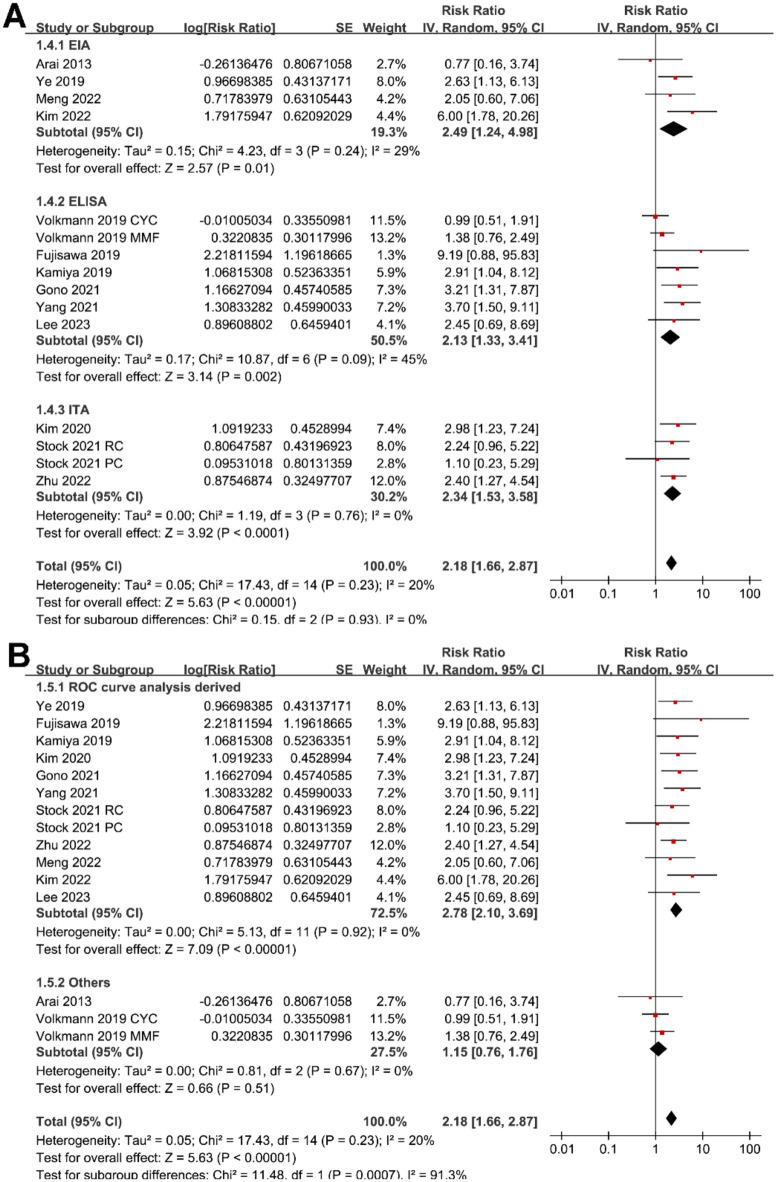
**Forest plots for the subgroup analyses of the association between serum KL-6 and mortality risk in patients with CTD-ILD.** (A) Subgroup analysis according to the methods for measuring serum KL-6 and (B) for defining the cutoff of KL-6. CTD-ILD: Connective tissue disease-associated interstitial lung disease; KL-6: Krebs von den Lungen-6; CI: Confidence interval; SE: Standard error.

**Figure 5.**
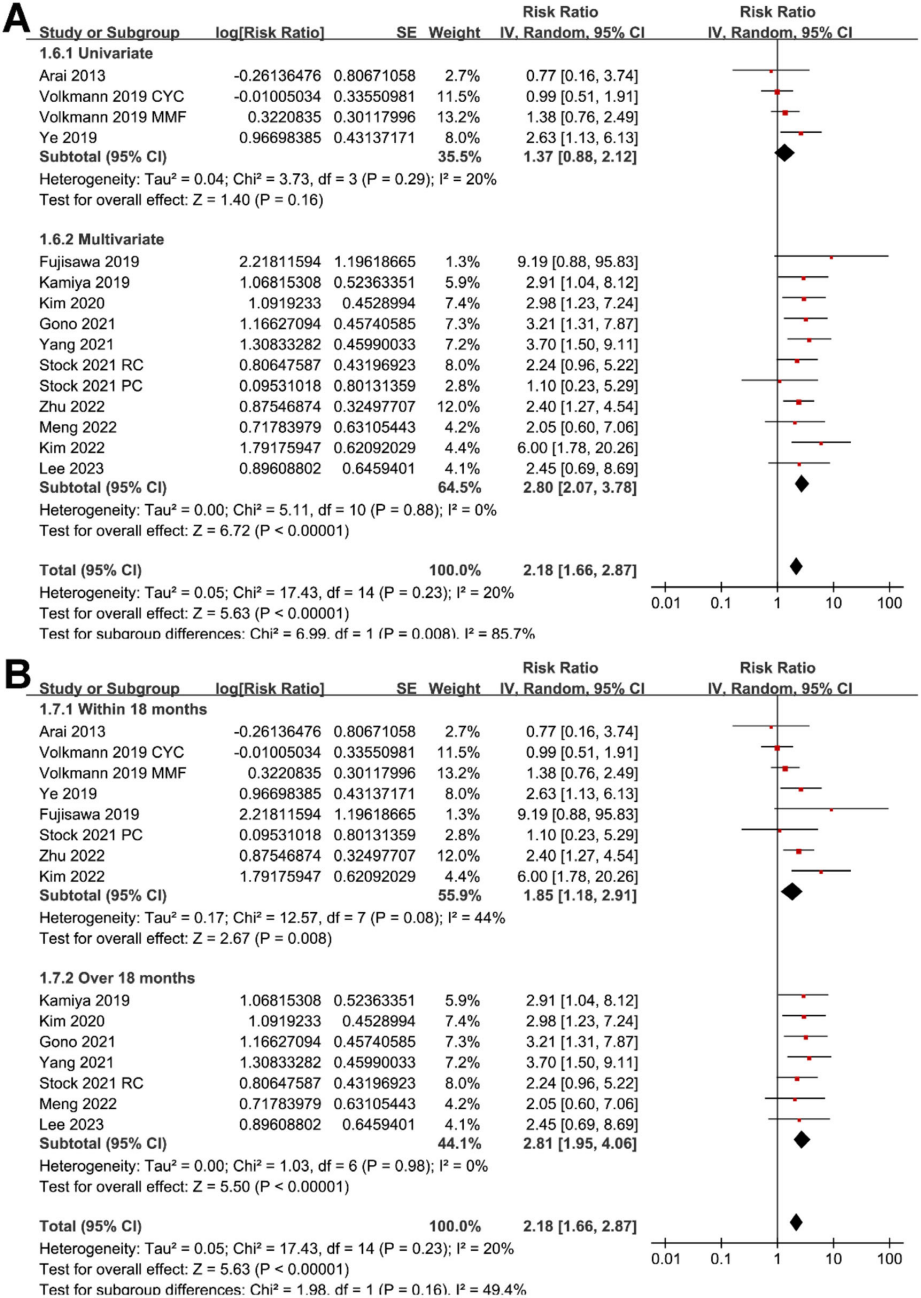
**Forest plots for the subgroup analyses of the association between serum KL-6 and mortality risk in patients with CTD-ILD.** (A) Subgroup analysis according to the analytic models and (B) subgroup analysis according the follow-up durations. CTD-ILD: Connective tissue disease-associated interstitial lung disease; KL-6: Krebs von den Lungen-6; CI: Confidence interval; SE: Standard error; CYC: Cyclophosphamide.

### Meta-analysis for the association between KL-6 and mortality of CTD-ILD patients

Pooled results with 15 cohorts [20–30, 39, 40] showed that compared to those with a lower KL-6 at admission, patients with a higher KL-6 were associated with a higher mortality risk during follow-up (RR: 2.18, 95% CI: 1.66–2.87, *P* < 0.001; Figure 2) with mild heterogeneity (*I*^2^ ═ 20%; Figure 2).

Sensitivity analysis by excluding one cohort at a time showed similar results (RR: 2.06–2.37, *P* all < 0.05). Subgroup analysis showed a significant association between serum KL-6 and mortality in patients with CTD-ILD in studies from Asian countries (RR: 2.82, 95% CI: 2.09–3.81, *P* < 0.001; *I*^2^ ═ 0%), but not in those from non-Asian countries (RR: 1.34, 95% CI: 0.92–1.96, *P* ═ 0.13; *I*^2^ ═ 0%; *P* for subgroup difference ═ 0.003; Figure 3A). Subgroup analysis according to the different types of CTD (*P* for subgroup difference ═ 0.10; Figure 3B) or different methods for measuring serum KL-6 (*P* for subgroup difference ═ 0.93; Figure 4A) did not significantly affect the results. Results of subgroup also suggested a significant association between high KL-6 and increased mortality in studies with cutoff of KL-6 derived from the ROC curve analysis (RR: 2.78, 95% CI: 2.10–3.69, *P* < 0.001; *I*^2^ ═ 0%), but not in those derived from median or upper limits of normal value (RR: 1.15, 95% CI: 0.76–1.76, *P* ═ 0.51; *I*^2^ ═ 0%; *P* for subgroup difference < 0.001; Figure 4B); in studies with multivariate analysis (RR: 2.80, 95% CI: 2.07–3.78, *P* < 0.001; *I*^2^ ═ 0%), but not in those with univariate analysis (RR: 1.37, 95% CI: 0.88–2.12, *P* ═ 0.16; *I*^2^ ═ 20%; *P* for subgroup difference ═ 0.008; Figure 5A). Subgroup analysis showed consistent results according to the different follow-up durations (*P* for subgroup difference ═ 0.16; Figure 5B).

### Publication bias evaluation

**Figure 6.**
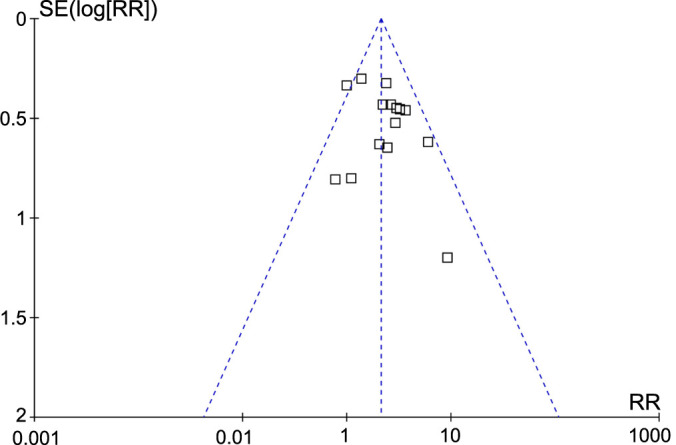
**Funnel plots for the publication biases underlying the meta-analyses of the association between serum KL-6 and the mortality risk in patients with CTD-ILD.** CTD-ILD: Connective tissue disease-associated interstitial lung disease; KL-6: Krebs von den Lungen-6; SE: Standard error; RR: Relative risk.

The funnel plots for the meta-analysis of the association between serum KL-6 and the mortality of patients with CTD-ILD are shown in Figure 6. The symmetrical nature of the funnel plots suggested the low likelihood of publication bias. Results of Egger’s regression test also showed a low risk of publication bias (*P* ═ 0.44).

## Discussion

In this study, we performed a meta-analysis by incorporating data of 15 cohorts, to examine the association between serum KL-6 and the mortality risk of patients with CTD-ILD. The results indicated that compared to the patients with a lower serum KL-6 at baseline, CTD-ILD patients with a higher serum KL-6 were associated with a higher risk of all-cause mortality during follow-up. Further sensitivity analysis by excluding one study at a time showed consistent results. In addition, subsequent subgroup analysis suggested that the association between a higher serum KL-6 and the increased mortality of CTD-ILD patients was mainly driven by Asian studies, studies with ROC curve analysis derived cutoff of serum KL-6, and studies with multivariate analysis. The association was not significantly affected by different CTDs, methods for measuring serum KL-6, of follow-up durations. In summary, the combined outcomes of this meta-analysis provide evidence for a plausible relationship between a higher serum KL-6 and the increased mortality of patients with CTD-ILD.

As far as we acknowledged, this study may be the first meta-analysis that summarized the association between serum KL-6 and the mortality risk of patients with CTD-ILD. Before the results of the meta-analysis are interpreted, it is essential to recognize the meticulous methodology employed in this meta-analysis. Notably, a thorough search of three widely utilized electronic databases was conducted, resulting in the identification of 15 contemporary cohorts that align with the objectives of this meta-analysis. Furthermore, only studies with longitudinal follow-up were considered, allowing for the examination of a sequential relationship between a higher serum KL-6 and the increased risk of mortality. Additionally, the robustness of the findings was further confirmed through various sensitivity and subgroup analyses, which suggested that the results were neither primarily driven by either of the included datasets nor they could be significantly affected by study characteristics such as different CTDs, methods for measuring serum KL-6, of follow-up durations. Collectively, the results of the meta-analysis underscore the significant association between serum KL-6 and increased mortality risk in patients with CTD-ILD, supporting the use of serum KL-6 as a prognostic factor in these patients. A previous meta-analysis of 29 studies showed that serum KL-6 had superior diagnostic accuracy to surfactant D for differentiating ILD from non-ILD among CTD patients [41]. These findings expanded the role of serum KL-6 for the management of patients with CTD-ILD, not only as a diagnostic biomarker but also as a prognostic predictor.

Interestingly, our subgroup analysis demonstrated a more remarkable association between serum KL-6 and mortality risk in studies of Asian countries as compared to those of Western countries. The underlying mechanisms for the potential ethnicity difference of the association between serum KL-6 and mortality risk in patients with CTD-ILD remains unknown. However, there are some studies that showed the increased prevalence, disease severity, and mortality of CTD-ILD in Asian patients. A recent systematic review included 34 studies that showed that the prevalence of ILD in patients with PM/DM was predominant in Asians [42]. A previous study from Canada that included 359 patients with CTD-ILD showed that Asian ethnicity was a predictor of decline in lung function and mortality in these patients [43]. Our subgroup analysis also showed that the association between serum KL-6 and the mortality risk of these patients was stronger in studies with a cutoff of KL-6 derived via the ROC curve analysis as compared to that derived via other methods such as the medians of KL-6. This is not surprising because the ROC curve analysis has been considered the standard method to retrieve the optimal cutoff value for prognostic prediction [44]. Finally, it is also suggested in subgroup analysis that a significant association between serum KL-6 and the mortality risk of these patients was mainly driven by studies with multivariate analysis rather than univariate analysis. These findings further support a potential independent association between serum KL-6 and mortality risk in these patients, which was not affected by confounding factors, such as the age and sex of the patients. This is important because advanced age has been recognized as a common risk factor for poor survival of patients with CTD-ILD, such as those with RA-associated ILD [45] and SSc-associated ILD [46].

The mechanisms underlying the association between serum KL-6 and mortality risk in patients with CTD-ILD may be explained by the physiological role of KL-6 as a biomarker of alveolar and bronchial epithelial cell injuries [10]. An increase in serum KL-6 has been related to reductions of forced vital capacity and diffusing capacity of the lung for carbon monoxide, two key parameters reflecting the lung functions [47, 48]. Accordingly, a higher serum KL-6 in patients with CTD-ILD is a marker of the severity of lung injury and impaired pulmonary function, indicating the progressiveness of the disease [49]. In addition, serum KL-6 could be conveniently and noninvasively measured in real-world clinical practice. All of these features enable serum KL-6 to be used as a potential prognostic predictor of patients with CTD-ILD.

This study also has some limitations to note. One important issue is that the cutoff value of serum KL-6 for the prediction of mortality risk in patients with CTD-ILD varied among the included studies, which may lead to between-study heterogeneity. Furthermore, substantial incorporated studies exhibited a retrospective design, thereby potentially subjecting the outcomes of the meta-analysis to recall and selection biases. To substantiate the findings of the meta-analysis, it is imperative to conduct extensive prospective cohort studies on a large scale. Moreover, we focused on the potential prognostic role of serum KL-6 in patients with CTD-ILD, while it was suggested that there are certain genetic factors that could also be involved in the association between ILD severity and CTD. For example, a recent study showed that the presence of the MUC1 rs4072037 C allele increases the risk of antisynthetase syndrome (ASS) and it could be a useful genetic biomarker for the differential diagnosis between ASS-ILD + and idiopathic pulmonary fibrosis patients [50]. The role of these genetic factors in the prediction of the mortality of patients with CTD-ILD could also be explored in future studies. Additionally, our study solely encompassed observational studies, thus precluding the establishment of a causal relationship between a high serum KL-6 and increased mortality risk of these patients. Finally, the meta-analysis was based on data from the study level rather than the individual-patient level. The results of the subgroup analyses should be cautiously interpreted and better validated in large-scale cohort studies.

## Conclusion

In conclusion, the findings from the meta-analysis suggest that compared to the patients with a lower serum KL-6 at baseline, CTD-ILD patients with a higher serum KL-6 were associated with a higher risk of all-cause mortality during follow-up. Although the mechanisms underlying the association between serum KL-6 and mortality risk in patients with CTD-ILD deserve further investigation and the results of the meta-analysis should be validated in prospective cohorts, these results highlight the potential use of serum KL-6 as a prognostic biomarker for patients with CTD-ILD.

## Data Availability

All data generated during the study are within the manuscript.
